# Pneumonia mortality and healthcare utilization in young children in rural Bangladesh: a prospective verbal autopsy study

**DOI:** 10.1186/s41182-018-0099-4

**Published:** 2018-05-25

**Authors:** Farzana Ferdous, Shahnawaz Ahmed, Sumon Kumar Das, Mohammod Jobayer Chisti, Dilruba Nasrin, Karen L. Kotloff, Myron M. Levine, James P. Nataro, Enbo Ma, Khitam Muhsen, Yukiko Wagatsuma, Tahmeed Ahmed, Abu Syed Golam Faruque

**Affiliations:** 1 Graduate School of Comprehensive Human Sciences, University of Tsukuba, Tsukuba, Japan; 2 International Centre for Diarrhoeal Disease Research, Bangladesh (icddr,b), Dhaka, Bangladesh; 3 School of Public Health, The University of Queensland, Brisbane, Australia; 4 Center for Vaccine Development and Department of Medicine, University of Maryland School of Medicine, Baltimore, MD USA; 5 Center for Vaccine Development, Department of Pediatrics and Medicine, University of Maryland School of Medicine, Baltimore, MD USA; 6 Department of Pediatrics, University of Virginia School of Medicine, Charlottesville, VA USA; 7 Health Promotion Center, Fukushima Medical University, Fukushima, Japan; 8 Department of Epidemiology and Prevention Medicine, School of Public Health, Sackler Faculty of Medicine, Tel Aviv, Israel; 9 Department of Clinical Trial and Clinical Epidemiology, Faculty of Medicine, University of Tsukuba, Tsukuba, Japan; 10 Nutrition and Clinical Services Division, icddr,b, 68 Shaheed Tajuddin Ahmed Sarani, Mohakhali, Dhaka, 1212 Bangladesh

**Keywords:** Death, Infant, Health care, Health facilities, Pneumonia

## Abstract

**Background:**

The present study aimed to examine the risk factors for death due to pneumonia in young children and healthcare behaviors of the guardians for children in rural Bangladesh. A prospective autopsy study was conducted among guardians of children aged 4 weeks to 59 months in Mirzapur, Bangladesh, from 2008 to 2012.

**Results:**

Pneumonia was the primary cause of death, accounting for 26.4% (*n* = 81) of all 307 deaths. Of the pneumonia deaths, 58% (*n* = 47) deaths occurred in younger infants (aged 4 weeks to < 6 months) and 24.7% (*n* = 20) in older infants (aged 6–11 months). The median duration of illness before pneumonia death was 8 days (interquartile range [IQR] 3–20 days). Prior to death, 91.4% (*n* = 74) children with pneumonia sought treatment, and of those who sought treatment, 52.7% (*n* = 39) sought treatment ≥ 2 days after the onset of disease. Younger infants of 4 weeks to < 6 months old were at 5.5-time (95% confidence interval [CI] 2.5, 12.0) and older infants aged 6–11 months were at 3-time (1.2, 7.5) greater risk of dying from pneumonia than older children aged 12–59 months. Children with a prolonged duration of illness (2–10 days) prior to death were at more risk for death by pneumonia than those who died from other causes (5.8 [2.1, 16.1]). Children who died from pneumonia sought treatment 3.4-time more than children who died from other causes. Delayed treatment seeking (≥ 2 days) behavior was 4.9-time more common in children who died from pneumonia than those who died from other causes. Children who died from pneumonia more often had access to care from multiple sources (5.7-time) than children who died from other causes.

**Conclusions:**

Delay in seeking appropriate care and access to multiple sources for treatment are the underlying risk factors for pneumonia death in young children in Bangladesh. These results indicate the perplexity in guardians’ decisions to secure appropriate treatment for children with pneumonia. Therefore, it further underscores the importance of focusing on mass media coverage that can outline the benefits of seeking care early in the progression of pneumonia and the potential negative consequences of seeking care late.

**Electronic supplementary material:**

The online version of this article (10.1186/s41182-018-0099-4) contains supplementary material, which is available to authorized users.

## Background

Pneumonia is the leading cause of childhood death, accounting for 16% of 5.6 million deaths of children aged less than 5 years globally, more than 95% of which occur in developing countries [1]. In Bangladesh, pneumonia accounts for 15% of the 119,000 total deaths of children aged less than 5 years in 2015 [2].

Numerous pathogens can cause pneumonia. The respiratory syncytial virus, *Streptococcus pneumoniae*, and *Haemophilus influenzae* are the leading causes of childhood pneumonia, with the latter two being preventable through vaccination [3]. These vaccines are currently included in the immunization programs of numerous countries. The main risk factors for pediatric pneumonia include not being breastfed, undernutrition, indoor air pollution, household crowding, low birth weight, incomplete immunization, HIV, and pre-existing illnesses such as underlying heart disease [4, 5]. Muscle weakness, soft rib abnormalities, chest wall deformities, and impaired immune function may increase the severity of the disease [6–8]. Cyanosis [9], inability to feed, malnutrition [9, 10], prolonged duration of illness [11], altered mental state [10], and the presence of underlying chronic illness (such as heart disease) [12] are related with increased pneumonia-associated mortality in young children.

In 2010, 48 million deaths, 7 million of which were children, occurred in low- and middle-income countries (LMICs), and most of these deaths occurred without medical attention, at homes in rural areas [13, 14]. It is commonly believed that child mortality is higher in rural rather than in urban areas [15]. It has been also reported that the post-discharge mortality due to pneumonia is as high as in-hospital mortality even after receiving successful treatment at hospital [16]. Parents living in remote areas have a lack of adequate knowledge about clinical features of pneumonia and do not perceive the illness as serious or life-threatening [17]. Although Bangladesh has achieved 80% immunization coverage, only 22% of children receive postnatal checkups for immunizations and only 37% receive facility treatment for acute respiratory infections (ARI) [18]. However, 12% of children do not receive any kind of treatment for ARI and a larger portion of ARI children do not receive facility treatment; their treatment forms do not yet reveal, where routine checkups have the potential to detect health problems and provide optimal health care [18]. Previous studies have highlighted prompt identification of symptoms or risk markers of pneumonia, and subsequent interventions may prevent complications and child deaths [19, 20]. Even with these suggestions and solutions, there are some underlying risks that have not yet come to light that require understanding and consideration to reduce the risk of death by pneumonia in children. Verbal autopsy is a technique of growing importance used to estimate the distribution of the cause of death in populations lacking vital registries or other medical death certificates [21]. The technique focuses on the child or maternal deaths to elicit information on the signs, symptoms, and sequence of events during the final illness leading to death, which has been increasingly used in LMICs [14, 22]. Therefore, this study aimed to examine the risk factors for deaths due to pneumonia in children less than 5 years of age in rural Bangladesh and describe patterns of healthcare utilization that preceded death.

## Methods

### Study design and study population

This prospective post-mortem verbal autopsy study was conducted from January 2008 to December 2012 within the framework of the Global Enteric Multicenter Study (GEMS). The present study comprised a secondary data analysis of risk factors of pneumonia mortality. The GEMS was conducted in four sites in sub-Saharan Africa (Gambia, Mali, Mozambique, and Kenya) and three sites in South Asia (India, Bangladesh, and Pakistan) and a rural sub-district (Mirzapur) which was the field site of the GEMS in Bangladesh [23, 24].

Mirzapur is a sub-district of the Tangail district located about 60 km north of Dhaka, the capital city of Bangladesh, and has a geographical area of 374 km^2^. The GEMS established a demographic surveillance system (DSS) in Mirzapur in January 2007 that covered eight out of 13 unions (the minimum administrative unit in rural Bangladesh). The DSS of GEMS regularly updated vital events including deaths in the study populations. In 2007, the mid-year population in the surveillance area was 238,463. The crude birth and death rates were 20.7 and 5.6 per thousand populations, respectively. The total fertility rate was 2388 per thousand women aged 15–49 years. The rate of neonatal mortality was 28.7 per thousand live births [25].

### Data collection—verbal autopsy

Verbal autopsies were conducted in case of any deaths of a child aged less than 5 years in the DSS area. The DSS of Mirzapur collected information on demographic vital events three times a year (every 4 months). The total area was divided into 15 clusters; one data collector was responsible for each cluster, and each cluster was further divided into 80 blocks. Each data collector visited a block (40–50 households) each day and covered 80 blocks within 16 weeks. Additionally, four data collectors were assigned to collect the most recent information on pregnancy outcomes. The database was updated on a weekly basis, and a list of recent deaths of children less than 5 years old, including neonatal deaths, was generated to conduct verbal autopsies. Eventually, an overall list of total deaths of children aged less than 5 years was obtained and the information was cross-checked with the weekly list generated on all deaths to detect any mismatches and/or to complete information that was not provided in the weekly list.

The causes of death were classified according to International Classification of Disease 10th edition (ICD-10) codes [26]. Information on the causes of death was collected in a standardized manner from the medical chart or healthcare providers and, if available, death certificate. The caretaker, typically the mother, was interviewed by a trained research assistant using a World Health Organization (WHO) standard verbal autopsy questionnaire 3 or 4 weeks after the child’s death [27]. Anonymous forms were reviewed by two clinicians independently to determine both the primary and antecedent causes of death of the child. In case of disagreement between the two clinicians, that was resolved through discussions with a third clinician. Detailed information on clinical signs and symptoms around the time of the child’s death, as well as care-seeking practices, were obtained. The questionnaire included both closed and open-ended questions.

#### Inclusion and exclusion criteria

The present study only focused on data collected by the WHO verbal autopsy questionnaire for children aged 4 weeks to 59 months. Two different WHO verbal autopsy questionnaires were used for data collection from two different age groups of children (0–28 days and 4 weeks to 59 months). Given the difficulty in matching data obtained from these two age groups for analysis, data collected from another WHO verbal autopsy questionnaire for neonates aged 0–28 days were excluded from present study analysis. We further excluded sepsis from the pneumonia risk factor analysis as severe cases of pneumonia can eventually lead to sepsis if not properly treated [28].

### Definitions

According to WHO classification, pneumonia was defined as the “presence of a cough and/or respiratory difficulty with any of the following symptoms reported by caregivers, such as first breathing, chest wall indrawing, noisy breathing, or flaring of nostril” [9].

In this study, the term “infant” denotes a child who was 4 weeks to 11 months old with children aged 4 weeks to < 6 months defined as “younger infants”, and children 6 to 11 months old are defined as “older infants.” The term “treatment” refers to any type of care including home remedies or other medical attention as reported by caregivers that were administered to the child with an illness that led to death. The term “indigenous healer” implies a person who is authorized by a particular culture or subculture to provide “indigenous treatment” even though he/she has not been so trained according to acceptable professional standards. The term, therefore, includes a whole range of individuals from *religious shamans*, *witch doctors*, and *medicine men* (*homeopathic*) within a community. The term “delay in treatment” represents a child who did not seek treatment within days of disease onset [29]. The term, therefore, considers a child with a delay of two and more days to seek treatment (median 1 day) for an illness that leads to death. “Malnutrition” denotes a child who had inadequate growth and thinness according to caregiver’s perception.

### Statistical analysis

Descriptive statistics were employed to describe the study sample and distribution of causes of death. The normality of the distribution was checked, and in case of a skewed distribution, median and interquartile range (IQR) values were given. Differences in the proportions of pneumonia death and other causes of death according to independent categorical variables were examined using the chi-square test. Crude and adjusted odds ratios (OR) and 95% confidence intervals (CI) were calculated for each independent variable. Age of the children was categorized into three groups considering younger infants at higher risk of death: (i) 4 weeks to < 6 months, (ii) 6 to 11 months, and (iii) 12 to 59 months (12–59 months was not further categorized due to insufficient frequency). Based on the median number of days to begin treatment after the disease onset, children were classified into three groups: (i) delayed in seeking treatment for ≥ 2 days, (ii) sought treatment within 0–1 day, and (iii) did not seek any treatment. The duration of disease that leads to death was categorized into three groups denoting prolonged duration of illness (> 10 days) at a higher risk of death: (i) 11 days and more, (ii) 2–10 days, and (iii) 0–1 day. Diagnosed medical conditions were classified into two groups based on presence or absence of any previous disease before the final illness that leads to death (yes/no). Before classifying diagnosed medical conditions into two groups, a cumulative frequency for all pre-existing diseases (congenital heart disease, malnutrition, kidney or liver diseases, cancer, asthma, and diarrhea) was estimated based on the presence or absence of the symptoms (yes/no). Symptoms noted during the final illness that leads to death were classified into two groups based on the presence or absence of any symptoms (yes/no). Before classification of symptoms into two groups, a cumulative frequency for all symptoms (diarrhea, vomiting, abdominal pain, no passage of stool, headache, presence of any mass, pain in neck, unconsciousness, convulsion, paralysis of lower limb, any change in urine flow, skin rash, red color of eyes, nasal bleeding, yellow color of urine, weight loss, thinness or wasting, and a change in hair color) was estimated based on the presence or absence of the symptoms (yes/no). Data from each source accessed for the treatment were collected separately. Responses from sources were merged into a separate variable, where government hospitals and government clinics were considered a single source, and as private hospitals, and private clinics were also considered as another single source of treatment.

Correlations between the independent variables were assessed using Spearman’s correlation coefficient. Highly correlated variables such as variables measuring healthcare utilization (correlation coefficients > 0.70) were analyzed in separate models to avoid multicollinearity among predictor variables. *P* < 0.10 in bivariate analysis was set as an inclusion criterion of variables in the multivariable models along with known predisposing factors such as age at death, previous illness diagnosed of the child, and symptoms noted during illness (any symptoms reported by mother) before death. Several multivariable logistic regressions were performed to identify the risk factors for pneumonia (ICD-10-CM code J18.8) as the primary cause, whereas other causes of death were used as a comparison group. In model 1, we included the following variables: seeking treatment before death (yes or no), age at death, preexisting diagnosed illness, symptoms, and duration of illness before death (symptoms which were likely to lead to death). An additional two models included variables relating to healthcare utilization and the interval between receiving treatment and death due to pneumonia. In model 2, all variables from model 1 were included except the variable “seeking treatment before death” which was replaced by another variable “sought care a number of days after onset of the disease.” In model 3, similar variables of model 2 were included, except “sought care a number of days after onset of the disease” variable was replaced by a variable denoting the “number of sources accessed to seek treatment.” *P* < 0.05 was considered as statistically significant. IBM SPSS (version 23.0; New York, USA) was used for data entry and analysis. Chi-square for linear trend was calculated by EpiInfo software (version 7.2).

## Results

**Fig. 1:**
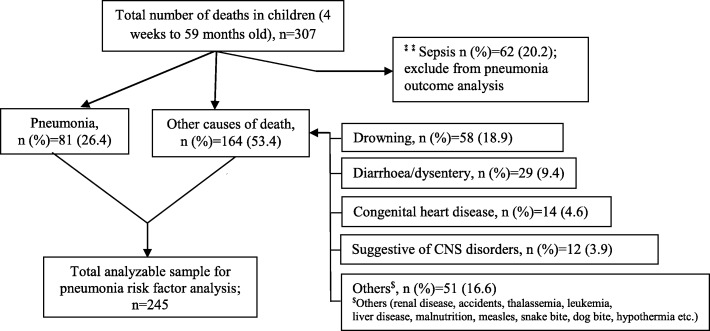
Flowchart of primary causes of death of children aged 4 weeks–59 months. ⁑⁑ To avoid overlapping clinical features, sepsis deaths (*n* = 62) were excluded from the analysis of risk factors for death from pneumonia

From January 2008 to December 2012, a total of 24,561 children were born in the DSS area of the Mirzapur and 307 (23.5/10,000) children aged 4 weeks to 59 months died during the study period. The cause-specific death rate for pneumonia (6.2/10,000) did not change during the study period (Table 1). The mean age at death was 14.6 months [standard deviation (SD) 15.3] (median 9.0 months). Approximately, 40.7% (*n* = 125) of all deaths occurred in younger infants (4 weeks to < 6 months), 16.9% (*n* = 52) occurred in older infants aged 6 to 11 months, and 42.3% (*n* = 130) in children 12 to 59 months old. Overall, 26.4% (*n* = 81) of all deaths were accounted for pneumonia. Other primary causes of deaths were sepsis (ICD-10-CM code A41.9) (20.2%; *n* = 62), drowning (18.9%; *n* = 58) (ICD-10-CM code V92), diarrhea/dysentery (9.4%; *n* = 29), congenital heart disease (4.6%; *n* = 14), and illness suggestive of central nervous system (CNS) disorders (3.9%; *n* = 12) (Fig. 1).

**Table 1:** Fertility and mortality indicators of rural Mirzapur DSS, 2008–2012

Indicators	2008	2009	2010	2011	2012	Overall	*p* value
Total population	239,233	244,754	258,061	263,085	264,998	239,233	
Total under-5 population	26,103	26,143	26,090	26,161	25,912	130,409	
Number of live births (CBR)	5176 (21.6)	4844 (19.8)	5040 (19.5)	5156 (19.6)	4345 (16.4)	24,561 (19.7)	0.0
Childhood deaths (4 weeks–59 months; CMR)	62 (23.8)	62 (23.7)	61 (23.4)	59 (22.6)	63 (24.3)	307 (23.5)	0.9
Childhood pneumonia deaths (4 weeks–59 months; CPMR) by under-5 population	21 (8.0)	11 (4.2)	16 (6.1)	16 (6.1)	17 (6.6)	81 (6.2)	0.8

Childhood death rate was measured as number of deaths per 10,000 total under-5 populations by each year period. Childhood pneumonia death rate was measured as number of pneumonia deaths per 10,000 under-5 population per year

*CBR* crude birth rate, *CMR* child mortality rate, *CPMR* childhood pneumonia mortality rate, *DSS* demographic surveillance system

To avoid overlapping clinical features, sepsis deaths (ICD-10-CM code A41.9) (*n* = 62) were excluded from the analysis of risk factors for death from pneumonia (Fig. 1). Of the children enrolled in the study (*n* = 245), 11.4% (*n* = 28) had a previous diagnosis of heart disease; 11% (*n* = 27) suffered from liver or kidney diseases, cancer, asthma, and diarrhea, etc.; and 4.9% (*n* = 12) had malnutrition. During the final illness that leads to death, fever was noted among 40% (*n* = 122) of the children and one third of them reported a cold and cough (*n* = 84), difficulty in breathing (*n* = 98), and chest indrawing (*n* = 75). The median duration of illness before child death was 4 days (interquartile range [IQR] 1–15 days) for all children, and for pneumonia deaths, the median duration of illness before death was 8 days (IQR 3–20 days). The median duration before seeking treatment after disease onset was 1 day (IQR 0–60 days [median 1.5 days for pneumonia]). For 66.5% (*n* = 163) of the children, treatment was sought before death, and of those who sought treatment, 67.5% (*n* = 110) children were admitted to a hospital or health facility for treatment and, for 43.6% (*n* = 71) children, treatment was sought ≥ 2 days after disease onset. Of those who sought treatment before death (*n* = 163), treatment was sought largely from private clinics/hospitals (79.7%; *n* = 130), followed by government facilities (28.2%; *n* = 46), pharmacies/drug stores (28.2%; *n* = 46), indigenous healers (16.6%; *n* = 27), and home treatment (6.1%; *n* = 10) (note: guardians accessed multiple sources for their child’s treatment; therefore, the figures do not add up to 100%).

### Risk factors for pneumonia deaths

Children aged 4 weeks to < 6 months, and those aged 6 to 11 months, when compared with older children 12 to 59 months old, were at increased risk to die from pneumonia than children who died from other causes. Children who died from pneumonia were ill for a longer period before death than those who died from other causes (53.2 vs. 21.4% were ill for 2–10 days, and 38 vs. 28.3% were ill for 11 days or more, respectively; *p* < 0.05). Children who died from pneumonia received treatment for their illnesses more often than those who died from other causes. Children who died from pneumonia were more likely to seek treatment ≥ 2 days after the onset of the illness than children who died from other causes (Table 2). Of the children with pneumonia who had visited three and more sources for treatment (*n* = 14), 71.4% (*n* = 10) of them delayed in seeking treatment until ≥ 2 days after the onset of disease and 75.0% (*n* = 11) of them had a duration of illness ≥11 days before death. Of those children with pneumonia who visited two sources for treatment (*n* = 24), 41.7% (*n* = 10) of them were delayed in seeking treatment ≥ 2 days after onset of disease and 58.3% (*n* = 14) of them had a duration of illness of 2–10 days before death (data not shown in the table).

**Table 2:** Correlates of pneumonia deaths compared to other causes of deaths in children aged 4 weeks to 59 months in rural Bangladesh (*n* = 245)

Variables	Pneumonia, *n* = 81 (%)	Other causes of death, *n* = 164 (%)	Unadjusted OR (95% CI)	Adjusted OR (95% CI)***
Age				
4 weeks–5 months	47 (58.0)	38 (23.2)	8.9 (4.4–18.0)*	5.5 (2.5–12.0)*
6–11 months	20 (24.7)	25 (15.2)	5.1 (2.6–13.0)*	3.0 (1.2–7.5)*
12–59 months	14 (17.3)	101 (61.6)	1	1
Sex				
Male	41 (50.6)	85 (51.8)	0.5 (0.6–1.6)	NA
Female	40 (49.4)	79 (48.2)	1	1
History of medical condition (multiple frequency)				
Heart disease	14 (17.3)	14 (8.5)	2.2 (1.0–5.0)*	1.4 (0.6–3.0)
Malnutrition	5 (6.2)	7 (4.3)	1.5 (0.5–4.8)	
Others (liver disease, kidney disease, cancer, asthma, diarrhea, etc.)	10 (12.3)	17 (10.4)	1.2 (0.4–2.8)	
None	52 (64.1)	126 (76.8)	1	1
Small at birth (< 1 year), *n* = 131^†^	21/67 (31.3)	16/64 (25.0)	1.4 (0.6–3.0)	NA
Premature (< 37 weeks) (< 1 year), *n* = 131^†^	15/67 (22.4)	4/64 (6.3)	4.3 (1.4–13.9)*	NA
Symptoms noted during last illness (multiple frequency)				
Fever	44 (54.3)	54 (32.3)	2.5 (1.4–4.3)*	0.9 (0.4–2.2)
Vomiting	13 (16.0)	34 (20.7)	0.7 (0.4–1.5)	
Not passed stool	5 (6.2)	22 (13.4)	0.4 (0.2–1.2)	
Unconscious	6 (7.4)	15 (9.1)	0.8 (0.3–2.1)	
Convulsion	9 (11.1)	17 (10.4)	1.1 (0.5–2.5)	
Others	39 (48.1)	67 (40.9)	1.3 (0.8–2.3)	
None	35 (43.2)	73 (44.5)	1	1
Duration of illness that leads to death (days), *n* = 238⁑				
≥ 11	30 (38.0)	45 (28.3)	7.6 (3.1–18.7)*	2.5 (0.8–7.4)
2–10	42 (53.2)	34 (21.4)	14.1 (5.8–34.6)*	5.8 (2.1–16.1)*
0–1	7 (8.9)	80 (50.3)	1	1
Sought care for illness that leads to death				
Yes	74 (91.4)	89 (54.3)	8.9 (3.9–20.5)*	3.4 (1.1–10.1)*
No	7 (8.6)	75 (45.7)	1	1
Sought care a number of days after onset of the disease (days)				
≥ 2	39 (48.1)	32 (19.5)	12.4 (5.0–30.7)*	NA
0–1	35 (43.2)	57 (34.8)	7.0 (2.9–16.7)*	
Did not seek treatment for illness	7 (8.6)	75 (45.7)	1	
Admission to a hospital/health facility, *n* = 163‡				
Yes	54 (73.0)	56 (62.2)	1.6 (0.8–3.2)	NA
No	20 (27.0)	34 (37.8)	1	
Sources of treatment (multiple frequency), *n* = 163‡				
Home	5 (6.8)	4 (4.5)	1.5 (0.4–5.9)	NA
Indigenous healer	12 (16.2)	15 (16.9)	0.9 (0.4–2.2)	
Government clinic/hospital	17 (23.0)	29 (32.6)	0.6 (0.3–1.2)	
Private clinic/hospital	62 (83.8)	68 (76.4)	1.6 (0.7–3.5)	
Pharmacies/drug stores	22 (29.7)	24 (27.0)	1.1 (0.6–2.3)	
Number of sources accessed to seek treatment				
≥ 3	14 (17.3)	15 (9.1)	10.0 (3.5–29.0)*	NA
2	24 (29.6)	30 (18.3)	8.6 (3.3–22.0)*	
1	36 (44.4)	44 (26.8)	8.8 (3.6–21.4)*	
0 (not sought treatment)	7 (8.6)	75 (45.7)	1	1
Place of death				
Hospital	33 (40.7)	29 (17.7)	3.2 (1.8–5.8)*	NA
Home	48 (59.3)	135 (82.3)	1	

Children who died from sepsis were exlcuded from the analysis

*OR* odds ratio, *CI* confidence interval, *NA* not applicable (those variables not included in the mutivariable model)

*p < 0.05

^†^Only available sample, *n* = 131

⁑Only available sample, *n* = 238

‡Only available sample, *n* = 163

***Model 1: dependent variable; causes of death (1 = pneumonia, 0 = other causes); variables included in the model were age at death, previous diagnosed illness (used as binominal variable—yes or no), symptoms noted during final illness (used as binominal variable—yes or no), duration of illness that leads to death, and sought care for illness that leads to death

Highly significant correlations (*r* > 0.7, *p* < 0.05) were found between three variables: seeking treatment, time elapsed (in days) between disease onset and seeking care, and the number of sources accessed for treatment (*r* = 0.869, 0.854, and 0.764, respectively) (Additional file 1: Table S1). As a result, these variables were not included in the same multivariable model.

Multivariable model 1 showed that younger infants aged 4 weeks to < 6 months were 5-time and older infants 6–11 months old were 3-time at increased risk of death for pneumonia than the older children 12–59 months old. Children with pneumonia had a prolonged duration of illness (6-fold more), particularly with duration of illness of 2–10 days than children who died from other causes. Children with pneumonia were more likely to seek treatment (3-fold more) than children who died from other causes (Table 2). The present study performed additional analyses (multivariable analyses) to identify knowledge gaps between care-seeking behavior and deaths from pneumonia. Multivariable model 2 showed that, in addition to age and duration of illness, children with pneumonia, who delayed ≥ 2 days from the onset of disease to seek care, were at more risk of death than the children who died from other causes. Multivariable model 3 showed that children with pneumonia were more likely to visit multiple sources (3 or more) to seek treatment than children who died due to other causes (Additional file 2: Table S2).

## Discussion

We assessed the risk factors for death due to pneumonia in children aged less than 5 years old in rural Bangladesh. Pneumonia was the leading cause of death found in 81/307 of all deaths of children aged 4 weeks to 59 months. Children who died from pneumonia were predominantly infants, with those aged less than 6 months being particularly at a higher risk. The present study revealed some important findings relating to death due to pneumonia in children and knowledge gaps in healthcare utilization by guardians of ill children in a rural community of Bangladesh. Firstly, only two thirds of these study children received treatment. Secondly, we noted that children who died from pneumonia had a prolonged duration of illness (median of 8 days) before death. Thirdly, a trend of delayed care seeking (≥ 2 days) after the onset of disease was noted in children who died from pneumonia rather than other causes. Lastly, children who died from pneumonia sought treatment from multiple sources more often before death than children who died from other causes. Altogether, these findings suggest that children who died from pneumonia had a time window between onset of symptoms and death, in which appropriate treatment could have been delivered to reduce the risk of death. However, in most cases, appropriate action was not taken during this critical timing. To our knowledge, this is the first study that posits delay in seeking care and access to multiple sources for treatment, as key factors for delaying appropriate care for pneumonia in children.

In seeking treatment, one single child was witnessed to utilize a maximum of four sources, including treatment at home (home remedy). This clearly indicates the reasons for delayed care seeking behavior for illness, which may be associated with families’ socioeconomic context and additional determining factors such as inadequate parental knowledge about the disease [17, 30]. The present study findings are consistent in some respects with previous studies as well as a WHO report which revealed the reasons for delayed seeking care (median 7 days) are treatment of children at home by the informal sector or indigenous healers, and such practices were important key barriers to prompt treatment and reduction of unnecessary childhood deaths due to pneumonia [20, 29]. In the present study, half of the children suffered at least 4 days due to the illness (for pneumonia, 8 days) and delayed at least 1 day (for pneumonia, 1.5 days) to seek treatment for the illness that leads to death. This lag time in between disease onset, treatment, and death of the children can be explained by the treatment sources those had been accessed by the guardians of ill children. Of those who sought treatment, one fourth (28.2%; 29.7% for pneumonia) of them attempted to seek treatment from pharmacies/drug stores, or 16.6% from indigenous healers (16.2% for pneumonia), or 6.1% received remedies at home (6.8% for pneumonia); additionally, 33.5% children never received any treatment for illnesses that lead to death (9% for pneumonia), suggesting inappropriate healthcare utilization patterns by guardians of sick children in this rural community of Bangladesh. It could be possible that guardians of pneumonia children accessed many different facilities, and due to the inappropriate or inadequate quality of care, their children could not recover and, subsequently, died. The present WHO verbal autopsy questionnaire for 4 weeks to 59 months old children could not reveal much meaningful information such as treatment quality at the facility and the places where the child was taken first to seek treatment.

According to UNICEF 2016 report, children younger than 2 years were at higher risk of death from pneumonia [31]; however, in the present study, infants (< 1 year old), especially less than 6 months old, were at a higher risk of death due to pneumonia than the older group. It has been reported that the immune system of children less than 6 months or 1 year old may be weakened if they are malnourished or if have not exclusively breastfed [32], though present study was unable to reveal those associations due to lack of those specific data. Former studies have indicated that guardians from rural as well as urban communities are unable to recognize the signs and symptoms of pneumonia, particularly the danger signs of pneumonia; thus, there is a delay in seeking care [17, 33]. In the present study, children with pneumonia often were reported as having symptoms such as fever, cough, and common cold. Diagnosis based on difficulty in breathing, chest indrawing, and noisy breathing (grunting or wheezing) can be easily performed to identify cases of childhood pneumonia or to measure the severity of disease. However, the present study findings indicate that caregivers were found not to pay careful attention to these symptoms at the time critical for ensuring appropriate treatment. Like other developing countries, the severity of a disease was not given due attention in rural Bangladesh at the initial stage, and parents tried to seek treatment from drug stores or local unlicensed healthcare providers [17, 20, 34]. These practices may have also occurred to the present study children. The advanced stage of the disease characterized by high fever, cough, difficulty in breathing, and first breathing may have influenced family members in the present study to take their children to the health facility, yet it appears guardians did not access them at the appropriate time. It has been also observed that 40% of children with pneumonia died at the hospital; however, 74% of children who died from pneumonia were admitted to hospitals to receive treatment for their illnesses. This gap between death and admission of children with pneumonia has been explained by Chisti et al. who reported that even after a successful treatment at a hospital, post-discharge mortality has been reported to be as high as in-hospital mortality [16] which suggests the possible existence of persistent subclinical infection even after recovery or the survivor of premature microbiota in these children.

Previous studies have reported that inadequate education, lack of decision-making in the family, poor household socioeconomic status, longer distance from healthcare facility, and inadequate knowledge about disease severity are the key barriers to accessing health care [17, 34–36]. Moreover, overall factors such as quality of care, costs, geographic barrier, and existing social network influence families to rely on community-level healthcare providers for their care [37]. Access to qualified healthcare providers to receive care is often impeded due to a lack of household income and the location of the family in a remote area, which is often accentuated by physical geographic barriers or the lack of transportation to reach the health facility in a timely manner [17, 37, 38].

Guardians of children living in areas similar to this study should be knowledgeable about child health and the advance signs and symptoms of common morbidities and further be aware of the appropriate measures necessary for prevention and early treatment. Moreover, programs, such as community-based treatment of childhood pneumonia, should consider the referral of children with complications to larger facilities for better treatment. A previous study in Zambia suggested health education in the rural community can help community members recognize clinically severe pneumonia and understand the importance of early and appropriate care seeking [39]. This study also proposes training and retraining of personnel in different levels of health care with an emphasis on case definition and case management, where management includes preventive measures and appropriate means of referral. Such training should prioritize the use of available protocols for health workers who play a key role in maternal and child health clinics, especially in the rural settings. By promoting appropriate care for young children, the mortality rate of children less than 5 years old would be reduced in Bangladesh, especially in the case of infants.

Limitations of the study

In the present study, all the information was collected on the basis of recall method (usually 3 to 4 weeks after death); to overcome this recall bias, trained research assistants carefully interviewed respondents which were again reviewed by research physicians. It is often accepted that details pertaining to stressful events like death are not easily forgotten; thus, authors believe these recalls as likely to be accurate [40]. Additionally, verbal autopsy questionnaires that were developed by WHO have some generic limitations due to lacking information on parent’s education, family income, the number of antenatal care (ANC) visits made by pregnant women, breastfeeding history, rate of breathing/minute, and the places where the first attempts to seek care were made. However, the likelihood of missing of data after conducting interviews was avoided by regular checks by the DSS personnel. Any mismatch or confusion on diagnosis was also avoided through quality training and reviewing of questionnaires by a group of trained and experienced physicians.

## Conclusions

Pneumonia is the number one killer in children less than 5 years of age in rural Bangladesh based on post-mortem verbal autopsies, and deaths mostly occur in infants. This study addresses important knowledge gaps by identifying factors such as caregivers’ delay in seeking appropriate care and access to multiple sources of treatment as the underlying risk factors for death due to pneumonia in young children in Bangladesh. The study findings suggest a time window between onset of symptoms and death for pneumonia, in which appropriate treatment can be delivered to reduce the risk of death. Prompt identification and treatment of less severe cases of pneumonia at the beginning may prevent their progression to severe pneumonia and death. A mass media program at the community level aimed at educating caregivers about the simple clinical features of pneumonia and intelligent utilization of health services might reduce childhood pneumonia deaths in Bangladesh. The government of Bangladesh should consider setting priorities and formulating appropriate strategies to educate the population to ensure improved healthcare behaviors which may address the basic need to improve health care in a resource-constraint setting like Bangladesh. Such an effort would achieve better results if both the public and the private sectors are involved in service delivery in rural Bangladesh.

## Additional files

Additional file 1: **Table S1.** Correlation between pneumonia and co-variates. (DOC 42 kb)

Additional file 2: **Table S2.** Correlates of pneumonia deaths compared to other causes of deaths in children aged 4 weeks to 59 months in rural Bangladesh. (DOC 56 kb)

## Abbreviations

ANC: Antenatal care
CI: Confidence intervals
CNS: Central nervous system
DSS: Demographic surveillance systems
GEMS: Global Enteric Multicenter Study
ICD-10: International Classification of Disease 10th edition codes
IQR: Interquartile range
LMICs: Low- and middle-income countries
OR: Odds ratios
SD: Standard deviation
UNICEF: The United Nations Children’s Fund
WHO: World Health Organization
