# Supplementary material for: Pneumonia mortality and healthcare utilization in young children in rural Bangladesh: a prospective verbal autopsy study
Source: Trop Med Health. 2018 May 25;46:17. doi: 10.1186/s41182-018-0099-4 (PMC5970515; doi:10.1186/s41182-018-0099-4)
Supplement: Supplementary file 1 — Table S1. Correlation between pneumonia and co-variates. (DOC 42 kb) [file 41182_2018_99_MOESM1_ESM.doc]

**Table S**1. Correlation between pneumonia and co-variates.

|  | Pneumonia | Child age | Previous medical condition | Symptoms noted during final illness (co-morbidity) | Duration of illness lead to death | Sought care for illness treatment | Sought care a number of days after onset of disease | Number of sources accessed to seek treatment |
| --- | --- | --- | --- | --- | --- | --- | --- | --- |
| Pneumonia | 1 | 0.419* | 0.113 | 0.239* | 0.299* | 0.370* | 0.382* | 0.302* |
| Child age |  | 1 | -0.020 | 0.240* | 0.369* | 0.374* | 0.336* | 0.282* |
| Previous medical condition |  |  | 1 | 0.193* | 0.419* | 0.262* | 0.310* | 0.309* |
| Symptoms noted during final illness (co-morbidity) |  |  |  | 1 | 0.457* | 0.537* | 0.440* | 0.488* |
| Duration of illness lead to death |  |  |  |  | 1 | 0.540* | 0.601* | 0.552* |
| Sought care for illness |  |  |  |  |  | 1 | 0.869* | 0.854* |
| Sought care a number of days after onset of disease |  |  |  |  |  |  | 1 | 0.764* |
| Number of sources accessed to seek treatment |  |  |  |  |  |  |  | 1 |

*p<0.05
