# Supplementary material for: Pneumonia mortality and healthcare utilization in young children in rural Bangladesh: a prospective verbal autopsy study
Source: Trop Med Health. 2018 May 25;46:17. doi: 10.1186/s41182-018-0099-4 (PMC5970515; doi:10.1186/s41182-018-0099-4)
Supplement: Supplementary file 2 — Table S2. Correlates of pneumonia deaths compared to other causes of deaths in children aged 4 weeks to 59 months in rural Bangladesh. (DOC 56 kb) [file 41182_2018_99_MOESM2_ESM.doc]

**Table S2** Correlates of pneumonia deaths compared to other causes of deaths in children aged 4 weeks to 59 months in rural Bangladesh

| **Factors** | | **aOR (95% CI)****^⁑^** | **aOR (95% CI)^⁑⁑^** |
| --- | --- | --- | --- |
| Age | |  |  |
|  | 4 weeks – 5 months | 5.7 (2.6 – 12.4)^*^ | 6.1 (2.7 – 13.7)^*^ |
|  | 6 – 11 months | 2.7 (1.1 – 6.9)^*^ | 3.2 (1.3 – 8.1) ^*^ |
|  | 12 – 59 months | 1 | 1 |
| Previous known medical condition | |  |  |
|  | Yes | 1.4 (0.6 – 2.9) | 1.5 (0.7 – 3.2) |
|  | No | 1 | 1 |
| Symptoms noted during final illness | |  |  |
|  | Yes | 1.1 (0.4 – 2.6) | 0.9 (0.4 – 2.2) |
|  | No | 1 | 1 |
| Duration of illness that leads to death (days) | |  |  |
|  | ≥11 | 1.9 (0.6 – 6.0) | 2.2 (0.7 – 6.8) |
|  | 2 – 10 | 5.0 (1.8 – 14.2)^*^ | 6.0 (2.2 – 16.8) |
|  | 0 – 1 | 1 | 1 |
| Health care utilization before death | |  |  |
|  | Sought care for illness that leads to death | NA | NA |
|  | Yes |  |  |
|  | No |  |  |
|  | Sought care a number of days after onset of the disease |  | NA |
|  | Care sought (≥2 days) | 4.9 (1.4 – 17.0)^*^ |  |
|  | Care sought (0-1 day) | 2.8 (0.9 – 8.7) |  |
|  | Did not seek treatment for illness | 1 |  |
|  | Number of sources accessed to seek treatment | NA |  |
|  | ≥3 |  | 5.7 (1.3 – 24.2) |
|  | 2 |  | 2.7 (0.8 – 9.4) |
|  | 1 |  | 3.6 (1.2 – 10.9) |
|  | 0 (not sought treatment) |  | 1 |

^*^p-value<0.05

**⁑**Model 2: Same variables of model 1 were used in the model 2, except variable,”sougth care for final illness that leads to death” replced by “sought care a number of days after onst of the diseaset”.

**⁑⁑**Model 3: Same variables of model 1 used in the model 3, except variable,”sougth care for final illness that leads to death” replced by “number of sources accessed to seek treatment ”.

Abberviations: aOR: Adjusted Odds Ratio; CI: Confidence Interval, NA: Not Applicable
